# Polymer Pro-Drug Nanoparticles for Sustained Release of Cytotoxic Drugs Evaluated in Patient-Derived Glioblastoma Cell Lines and In Situ Gelling Formulations

**DOI:** 10.3390/pharmaceutics13020208

**Published:** 2021-02-03

**Authors:** Catherine E. Vasey, Robert J. Cavanagh, Vincenzo Taresco, Cara Moloney, Stuart Smith, Ruman Rahman, Cameron Alexander

**Affiliations:** 1 School of Pharmacy, University of Nottingham, Nottingham NG7 2RD, UK; catherine.vasey@nottingham.ac.uk (C.E.V.); robert.cavanagh@nottingham.ac.uk (R.J.C.); vincenzo.taresco@nottingham.ac.uk (V.T.); 2 School of Chemistry, University of Nottingham, Nottingham NG7 2RD, UK; 3 School of Medicine, BioDiscovery Institute-3, University Park, Nottingham NG7 2RD, UK; cara.moloney@nottingham.ac.uk (C.M.); stuart.smith@nottingham.ac.uk (S.S.); 4 School of Medicine, Queen’s Medical Centre, Nottingham NG7 2UH, UK

**Keywords:** brain tumour, local delivery, polymer pro-drug, nanoparticles, doxorubicin

## Abstract

Glioblastoma (GBM) is the most common, malignant and aggressive brain tumour in adults. Despite the use of multimodal treatments, involving surgery, followed by concomitant radiotherapy and chemotherapy, the median survival for patients remains less than 15 months from diagnosis. Low penetration of drugs across the blood-brain barrier (BBB) is a dose-limiting factor for systemic GBM therapies, and as a result, post-surgical intracranial drug delivery strategies are being developed to ensure local delivery of drugs within the brain. Here we describe the effects of PEGylated poly(lactide)-poly(carbonate)-doxorubicin (DOX) nanoparticles (NPs) on the metabolic activity of primary cancer cell lines derived from adult patients following neurosurgical resection, and the commercially available GBM cell line, U87. The results showed that non-drug-loaded NPs were well tolerated at concentrations of up to 100 µg/mL while tumour cell-killing effects were observed for the DOX-NPs at the same concentrations. Further experiments evaluated the release of DOX from polymer-DOX conjugate NPs when incorporated in a thermosensitive in situ gelling poly(DL-lactic-co-glycolic acid) and poly(ethylene glycol) (PLGA/PEG) matrix paste, in order to simulate the clinical setting of a locally injected formulation for GBM following surgical tumour resection. These assays demonstrated drug release from the polymer pro-drugs, when in PLGA/PEG matrices of two formulations, over clinically relevant time scales. These findings encourage future in vivo assessment of the potential capability of polymer–drug conjugate NPs to penetrate brain parenchyma efficaciously, when released from existing interstitial delivery systems.

## 1. Introduction

Glioblastoma (GBM) is the most common, malignant and aggressive brain tumour in adults, mainly due to its rapid proliferation and ability to penetrate and diffusely infiltrate healthy brain parenchyma [1]. Standard of care treatment currently involves a combination of surgery, radiotherapy and chemotherapy. Yet, despite this multimodal treatment method, the median survival remains less than 15 months from diagnosis [2]. Local, intracranial drug delivery has been developed over the last two decades in order to exploit the low transport of drugs across the BBB, and thus ensure that locally delivered drugs remain in the brain [3]. As surgery is the usual first step in the treatment of GBM, the rationale for intracranial drug delivery is based upon a unique treatment window immediately adjuvant to neurosurgery, in this case by delivering drugs directly to the site of minimal volume residual tumour following surgery. In addition, the time just after surgery may be when tumour heterogeneity is at a minimum, thus facilitating drug treatment with lower possibilities of resistance. However, more efficacious treatment methods are still necessary to eradicate residual tumour cells which remain beyond the resection cavity lining after surgery, as GBM recurrence is almost certain unless these cells are eradicated [4].

Recently, a poly(DL-lactic-co-glycolic acid) and poly(ethylene glycol) (PLGA/PEG) matrix, consisting ofa thermosensitive in situ gelling PLGA/PEG microparticles, has been developed as a localised drug delivery system for the treatment of GBM [5,6]. The microparticles are mixed with saline solutions at room temperature to form a paste, enabling a surgeon to line the resection cavity site following removal of the tumour. The microparticles then sinter at 37 °C when the microparticles fuse, retaining the cavity lining shape. Initial studies have demonstrated that chemotherapeutic drugs can be embedded into the drug delivery matrix and released gradually from the original resection site in the brain, thus efficaciously targeting the residual infiltrative cells remaining beyond the resection site with significant overall benefits observed in an orthoptic pre-clinical model [7]. Localised drug delivery for GBM in this way has major advantages over delivering drugs systemically, mainly that high therapeutic drug concentrations can potentially be achieved at the malignant tumour site, whilst avoiding systemic dose-limiting side effects. This method has been shown to control and sustain the release of chemotherapeutic drug(s) over several weeks, thus filling the current oncological treatment gap between surgery and radiotherapy/chemotherapy (typically 3–4 weeks post-surgery).

However, penetration of therapeutic agents from the delivery depot to brain cells beyond the initial layers at the resection margin still presents a major challenge to effective treatment. Transport of drugs from the depot takes place largely via passive diffusion, and this can lead to non-specific uptake of the drugs in healthy cells, as well as the intended cancer cells, thus limiting further penetration of the drug into underlying cancerous tissue. In turn, this can result in regrowth of the cancer from beneath the resected layer. There is accordingly a need to transport the drugs to reach this layer, and the use of a nanoparticulate carrier is one means by which this might be achieved.

NPs can be internalised by two major endocytosis pathways: phagocytosis and pinocytosis [8]. Pinocytosis accounts for the cellular uptake of smaller NPs (<500 nm) and can be further distinguished by clathrin-dependent and clathrin-independent pathways [9,10]. NPs can be released from cells via exocytosis, which is distinguished by two main mechanisms: regulated exocytosis and constitutive exocytosis. Regulated exocytosis occurs when the contents of an intracellular vacuole are secreted from the cell in response to a specific signal, whereas constitutive exocytosis does not require a signal to secrete the macromolecules [11,12]. Cell internalisation and exocytosis pathways are of importance when considering the design and application of drug-loaded nanocarriers [13], and repeated endocytic and exocytic processes might be exploited by appropriately-designed NPs to enable ‘hopping’ of the carriers through multiple cell layers in order to enhance penetration through brain parenchyma. The conjugation of drugs with polymers to form so called polymeric pro-drugs is a promising approach to overcome drug limitations, such as solubility and stability, as well as encoding for drug release in specific disease environments [14,15].

We have recently reported polymer pro-drugs based on a PEGylated poly(lactide)-poly(carbonate) copolymer (mPEG_n_-LA_m_-TBPC_p_) [16,17], which is based on well-established chemistries and components already in clinical use. The polymer contains a carbamate moiety, in the side-chains of the monomer units, which after simple deprotection chemistry generates a free primary amine functionality, offering a simple conjugation point for drugs to be linked to the polymer backbone [16,17]. The polymer contains primary amine functionality in the side-chains of one of the monomer units, offering a simple conjugation point for drugs to be linked to the polymer backbone. We selected DOX as the drug candidate of first choice, due its use in multiple studies in vitro [18], in vivo [19] and human clinical trials [20,21], and its relevance in the treatment of GBM. Systemic injection of free DOX, however, does not lead to high concentrations crossing the blood–brain barrier (BBB) [22], and so many researchers have utilised DOX-loaded NPs as a means to traverse the BBB [23,24,25]. For our studies, we wanted to evaluate whether localised delivery of polymer-DOX pro-drug NPs from the PEG-PLGA in situ gelling paste could avoid the issues of BBB transport while also allowing for transcytotic ‘hopping’ [26] of the NPs beyond the resection layer. In this way, we aimed to enhance the delivered dose, while also increasing the potential targeting of DOX to cancerous cells by conjugating it to the polymer by a urea linker, which could exploit the higher urease activity reported in some cancer cells. We further aimed to address the increased intrinsic resistance of GBM to chemotherapeutic drugs, caused by the heterogeneity of the tumour micro-environment and subsequent genetic and epigenetic variation in tumour subclones as a result of variable selective pressures. Most commercial GBM cell lines have been historically derived from the MRI contrast-enhanced core region of tumours, which does not allow a realistic, phenotypically accurate representation of the infiltrative cells which remain after surgery and ultimately result in the inevitable recurrence of GBM [27]. Significantly, residual cells at the tumour margin are responsible for the ~85% of GBMs that relapse locally (typically within 2 cm^3^ of the infiltrative margin) after maximal safe surgical resection followed by the standard combination protocol of oral temozolomide and radiotherapy [28]. Thus, we wanted to evaluate whether the polymer pro-drugs designed to transport and release DOX might be more active against invasive patient-derived GBM cells than the standard GBM cell lines, as this would be an important step for developing more clinically-relevant therapies for this pathology [29]. We therefore utilised cell lines derived from the infiltrative margin of adult patients, defined by areas of minimal fluorescence during 5-aminolevulinic acid (5-ALA) fluorescence-guided neurosurgical resection, at the Queen’s Medical Centre, University of Nottingham [7,29]. We have thus studied the effects of polymeric pro-drug NPs on the metabolic activity of these primary cell lines and the widely-used commercial GBM cell line, U87. In addition, we have studied the release profile of the DOX-loaded NPs from the PLGA/PEG matrix and DOX release from NPs as the first steps towards establishing the dual polymer pro-drug/depot delivery system for GBM therapy.

## 2. Materials and Methods

All materials were used as obtained unless otherwise stated. Dulbecco’s Phosphate-Buffered Saline (PBS; modified without calcium chloride and magnesium chloride; sterile-filtered), Dimethyl Sulfoxide (DMSO, >99.7%; sterile-filtered) and sterile water (sterile-filtered) were purchased from Sigma Life Science (St. Louis, MO, USA). Astrocyte Medium (AM; sterile filtered), Poly-L-Lysine (1 mg/mL; sterile filtered), Trypsin/EDTA solution (T/E; 0.25%; sterile-filtered) and Trypsin Neutralisation Solution (TNS; sterile filtered) were purchased from ScienCell Research Laboratories (Carlsbad, CA, USA). Hank’s Balanced Salt Solution (HBSS; sterile-filtered), Dulbecco’s Phosphate-Buffered Saline (DPBS; supplemented with calcium chloride and magnesium chloride), Dulbecco’s Modified Eagle Medium (DMEM; supplemented with 1 g/L D-glucose L-glutamine and pyruvate), phenol red-free DMEM (with 4500 mg/L glucose and sodium bicarbonate and without L-glutamine, sodium pyruvate and phenol red) and FluoroBrite DMEM were purchased from Gibco^TM^ by ThermoFisher Scientific (Waltham, MA, USA). PrestoBlue^TM^ Cell Viability Reagent and Hoechst 33342 were purchased from Invitrogen^TM^ by ThermoFisher Scientific. HyClone^TM^ Bovine Growth Serum (FBS; GE Healthcare) was purchased from Fisher Scientific by ThermoFisher. Acetone (>99.8%) was purchased from Fisher Chemical (Hampton, NH, USA). Dimethyl Sulfoxide (DMSO) was purchased from Honeywell (Charlotte, NC, USA). Poly(ethylene glycol) (PEG; M_n_ = 400 Da) was purchased from Aldrich Chemistry. Poly(lactic-co-glycolic acid) Resomer^®^ (P_DL_LGA; Select 85:15 DLG 4CA; technical grade) was purchased from Evonik (Essen, Germany). Doxorubicin (hydrochloride) (DOX.HCl) was purchased from Cayman Chemical company (Ann Arbor, MI, USA). Synthesis of all the polymers was via ring opening of lactide and a t-butyloxycarbamoyl-protected cyclic carbonate monomer (TBPC) derived from serinol by methoxypoly(ethyleneglycol)s to generate polymers abbreviated as mPEG_5000_-(LA)_n_-(TBPC)_y_, followed by Cy5-labelling and DOX coupling to produce the final pro-drug platforms were performed following previously reported protocols [16,17].

### 2.1. Nanoparticle Stability Studies

NPs were formed using a ‘nanoprecipitation’ method to generate kinetically trapped, ‘micellar-like’ NPs. In these experiments, the polymers (10 mg) were dissolved in acetone (1 mL), and the polymeric dispersion was added dropwise to deionised water (10 mL, final concentration of 1 mg/mL) under constant stirring at 550 rpm. The polymers rapidly formed NP suspensions as solvent exchange between water and acetone led to aggregation of the hydrophobic poly(lactide-co-carbonate) blocks. The final suspension was subsequently dialysed in a 3.5 kDa molecular weight cut-off dialysis tubing against PBS overnight in order for complete exchange between water and PBS. The resultant sizes of the NPs were derived via DLS measurements following filtration using a 0.22 µm filter. Control NPs (final conc. 500 µg/mL) were stored at 4 °C, whilst another set were mixed 1:1 with DMEM (final conc. 500 µg/mL) and stored at 37 °C, to provide a cell culture medium environment. The particle sizes were determined by DLS at time points 1, 4, 24, 48 and 168 h using a Zetasizer Nano ZS (Malvern Instruments Ltd., Malvern, UK). Measurements were taken in triplicate of NPs suspensions at 500 µg/mL and used to calculate mean intensity particle size distributions.

### 2.2. Cell Culture

The U87 GBM cell line was obtained from ATCC and cultured within a passage window of 13 passages. U87 cells were cultured in Dulbecco’s Modified Eagle Medium (DMEM) supplemented with 10% (*v*/*v*) Foetal Bovine Serum (FBS) at 37 °C in a humidified incubator with 5% CO_2_. GIN-8 (Glioma INvasive margin cells) were isolated from medial front invasive margin (54 y female, wild-type IDH (primary GBM), intact ATRX, 0% MGMT promoter methylation, 90% resection plus Gliadel wafers; treatment 60Gy radiotherapy, concurrent and adjuvant temozolomide; patient died 5 months after surgery), GIN-28 were isolated from 5-ALA fluorescence-positive invasive margin (71 y male, wild-type IDH (primary GBM), intact ATRX, 0% MGMT promoter methylation 99% resection; no adjuvant therapy (patient choice); died 3 months after surgery) [29] and GIN-31 were isolated from 5-ALA fluorescence-positive invasive margin (wild-type IDH (primary GBM), intact ATRX, 0% MGMT promoter methylation, 100% resection; treatment 60Gy radiotherapy, concurrent and adjuvant temozolomide; patient died 16.1 months after surgery) by our laboratory, and were used at passages of 24–50, 26–41 and 28–55, respectively. GIN cell lines were cultured in Dulbecco’s Modified Eagle Medium (DMEM; Sigma-Aldrich, St. Louis, MO, USA) supplemented with 10% FBS, 1 g/L glucose and 2 mM L-glutamine (Sigma-Aldrich) at 37 °C with 5% CO_2_. The optimisation of seeding densities for the GIN lines for assaying was based on their proliferation and is shown in Supplementary Figure S1. A primary human astrocyte cell line was used as a comparator of healthy brain cells. This was obtained from ScienCell Research Laboratories (catalogue #1800) and cultured between passage 3 and 10. Primary human astrocytes were cultured in Astrocyte Medium (AM) supplemented with 2% FBS (*v*/*v*), 1% penicillin-streptomycin (*v*/*v*) and 1% Astrocyte Growth Medium (*v*/*v*) at 37 °C in a humidified incubator with 5% CO_2_. Cells were cultured in flasks previously coated with poly-l-lysine (150 µL; 10 mg/mL) and sterile water (10 mL; final concentration 2 µg/cm^2^) for a minimum of one hour to promote adhesion of the cells. The 9L rat glioma cell line was received as a gift from Prof. Henry Brem and Dr. Betty Tyler from Johns Hopkins University. The cell line was cultured in DMEM supplemented with 10% FBS, 1 g/L glucose and 2 mM L-glutamine at 37 °C with 5% CO_2_.

### 2.3. Cytotoxicity Experiments

The PrestoBlue™ Cell Viability assay was performed to assess the metabolic activity of cell lines used following exposure to blank NPs, doxorubicin and doxorubicin-NPs. All cells were seeded at a density of 5 × 10^3^ cells per well, with the exception of 9L and GIN-31 cells which were seeded with 3 × 10^3^ cells per well, in 96 well plates and cultured for 24 h prior to treatment. Cells were exposed to treatments for 72 h and applied in 100 µL DMEM containing 10% (*v*/*v*) FBS. DMSO (100%) was used for a cell death (positive) control and a vehicle control (100% DMEM) used as a negative control. Following exposure, treatments were removed, and the cells were subsequently washed twice with warm Hank’s Balanced Salt Solution (HBSS). A volume of 110 µL 10% (*v*/*v*) PrestoBlue™ reagent diluted in HBSS was applied per well for 45 min. The resulting fluorescence was measured at 544/590 nm (λex./λem.) on a BMG Labtech FLUOstar Omega microplate reader. Relative metabolic activity was calculated by setting normalised values from the negative control as 100% and positive control as 0% metabolic activity.

### 2.4. Fluorescent Live-Cell Microscopy

To investigate cellular uptake, live-cell fluorescence microscopy was used to image GIN8, GIN28 and U87 cells. NPs for uptake experiments were prepared as previously described but were formulated as 10% *wt/wt* of mPEG_5000_-(LA)_50_-(TBPC)_50_-Cy5 (P2-Cy5) and 90% *wt/wt* mPEG_5000_-(LA)_50_-(TBPC)_50_ (P2) and diluted to a final concentration of 50 µg/mL in 10% (*v*/*v*) FBS containing DMEM (no phenol red). GIN8, GIN28 and U87 cells were seeded in CellView™ 35 mm diameter glass-bottom cell culture dishes at a density of 2.5 × 10^5^ cells per dish and cultured for 24 h. Cy5-NPs (50 µg/mL) were incubated with cells for 0.5, 2 and 4 h at 37 °C with 5% CO2. Following exposure, NP solutions were removed and cells washed three times with ice-cold PBS. Cells were then stained with 10 µg/mL Hoechst 33342 (Thermo-Fisher) or 10 µg/mL Hoechst 33342 and 50 nM Lysotracker green DND-26 (Thermo-Fisher) applied in Hank’s Balanced Salt Solution (HBSS) for 30 min. Staining solution was removed and cells washed twice with PBS. FluoroBrite DMEM was added to wells and cells were imaged on an inverted Nikon Eclipse TE 300 fluorescent microscope on DAPI, FITC and Cy5 filters. Images were processed using ImageJ software (1.52f) and the Coloc 2 plug in was used for calculation of Pearson’s correlation coefficient for co-localisation studies.

### 2.5. Fluorometric-Based Uptake Assessment

GIN8, GIN28 and U87 cells were plated at 5.8 × 10^4^ cells per well in 24 well plates. Following a 24 h culture period, culture medium was removed and Cy5-NPs were applied in phenol red free medium containing 10% (*v*/*v*) FBS. The time dependence of cell uptake was assessed with 50 μg/mL solutions of nanoparticles and at time points of 5, 15, 30, 60, 120, 180, and 240 min. Following exposure, NP solutions were removed and cells washed three times with ice-cold PBS. Cells were then permeabilised with 1% (*v*/*v*) Triton X-100 solution applied in PBS for 10 min at 37 °C. Permeabilised cells were thereafter pelleted by centrifugation and nanoparticles quantified by fluorescent measurement at 640/680 nm (λex./λem.) using a Tecan Spark 10M plate reader. Quantification of nanoparticle uptake was achieved via calibration curves of known nanoparticle concentrations diluted in 1% (*v*/*v*) Triton X-100 in PBS solution. Values were normalised to viable cell number per well determined by the trypan blue exclusion test and cell counting on a haemocytometer.

### 2.6. Preparation of PLGA/PEG Matrix

The PLGA/PEG paste was prepared as described previously [30,31]. In brief, thermosensitive microparticles were fabricated from blends of 53 kDa P_DL_LGA (85:15 DLG 4CA) and PEG. A mixture of 93.5:6.5% PLGA:PEG (*w*/*v*) was blended at 80–90 °C on a hotplate. The melted PLGA and PEG were mixed by hand using a polytetrafluoroethylene (PTFE)-coated spatula and the mixture cooled to room temperature. Polymer blend sheets were then ground into particles in a bench-top mill (Krups Mill F203) and the particles were sieved to obtain the 100–200 µm particle size fraction.

### 2.7. Release Studies

#### 2.7.1. DOX Release from DOX-NPs

Polymer-DOX conjugates were formulated by nanoprecipitation to form DOX-NPs of size 67 nm and at a concentration of 2 mg/mL in PBS, as previously described, and DOX release from DOX-NPs was measured. Following dialysis against PBS, the resultant NPs (500 µL) were pipetted into a Slide-A-Lyser^®^ MINI Dialysis device with a 3.5 kDa molecular weight cut off, with 14 mL PBS in the Eppendorf below. At set time points (*t* = 0.02, 0.04, 0.08, 0.17, 0.25, 1, 2, 3, 6, 7, 28 and 44 days), the entire 14 mL PBS sample was removed and replaced with fresh PBS. The removed PBS sample was freeze-dried and resuspended in DMSO (5 mL), sonicated for 10 min and centrifuged for a further 10 min. The DOX present in the resultant supernatant sample (2 mL) was analysed by fluorescence on a Varian CaryEclipse Fluorescent Spectrometer. The sample was read on the slow scan mode, 450/510–640 nm (λex./λem.) at 900 V, with excitation split set at 5 nm and emission split set at 10 nm.

#### 2.7.2. DOX-NP Release from PLGA/PEG Paste

DOX-NPs were formulated as previously described (5 mg/mL). When forming the PLGA/PEG paste, the DOX-NP solution replaced the PBS to form DOX-NP-loaded-PLGA/PEG paste. PLGA/PEG microparticles (636 mg; 100–200 µm) were mixed with 509 µL DOX-NPs (dialysed overnight against PBS; final conc. 2.65 mg/mL) and were split evenly six ways (106 mg PLGA/PEG; 84.83 µL DOX-NP), approximately maintaining the previously optimised 1:0.8 (polymer:saline) ratio required for a glass transition temperature of 37 °C [6]. Three formulations were packed tightly into cylindrical moulds (6 × 6 × 2 mm; l × w × d) and the remaining three were pasted lightly into a weighing boat, producing a thin layer to mimic more closely how the polymer will be applied to a resection cavity in vivo. The PLGA/PEG matrices were sintered overnight at 37 °C. Release was assessed through placing the sintered PLGA/PEG matrices into a scintillation vial containing 5 mL PBS. At set time points (*t* = 0.02, 0.04, 0.08, 0.17, 1, 2, 5, 6, 7, 28, 43 and 63 days), the entire 5 mL PBS sample was removed and replaced with fresh PBS. The PBS sample was freeze dried, resuspended in DMSO (5 mL), sonicated for 10 min before centrifuging for a further 10 min. The DOX present in the resultant supernatant sample (2 mL) was analysed by fluorescence spectroscopy using a Varian CaryEclipse Fluorescent Spectrometer and was, by proxy, attributed to DOX-NP release from PLGA/PEG paste. The sample was read on the slow scan mode, 450/510–640 nm (λex./λem.) at 900 V, with excitation split set at 5 nm and emission split set at 10 nm.

### 2.8. Statistical Analysis

Except where stated otherwise, in the individual experimental methods sections above, each in vitro experiment was performed independently three times in triplicate. Statistical significance was accepted at a level of *p* < 0.05 (* *p* < 0.05; ** *p* < 0.01; *** *p* < 0.001; **** *p* < 0.0001). GraphPad Prism software (v8.1) was used for statistical analysis and IC_50_ values calculated by fitting the data to a non-linear dose–response curve with a variable slope.

## 3. Results

**Scheme 1:**
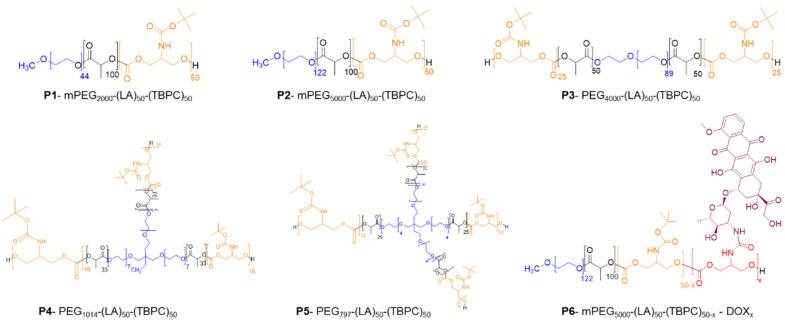
Polymers synthesised for initial evaluation as drug carriers.

The polymers used in this study are shown in Scheme 1 and their key attributes in Table 1.

The polymers were all synthesised by ring-opening polymerisation, as described previously [16,17], and were characterised by NMR spectroscopy and gel permeation chromatography (GPC), as shown in Table 1.

### 3.1. Nanoparticle Stability

Initial stability studies were carried out in order to mimic two common environments for nanoformulations, in particular, “control NPs” were kept at 4 °C to replicate storage of NPs in standard cool-chain conditions and the “experimental NPs” were kept at 37 °C in DMEM, replicating the physiological conditions for future in vitro work. A DLS-based assay was chosen as the screening technique due to the fast response time and ease of detection of aggregation within each sample [32].

**Figure 1:**
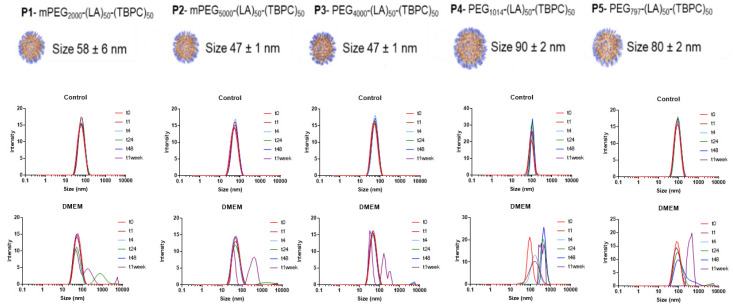
Dynamic Light Scattering (DLS) plots for NP formulations of P1–P5 under cool-chain storage (‘control’) conditions (PBS at 4 °C; top row) and in cell culture medium (DMEM at 37 °C; bottom row) after 0, 1, 24, 48 h and 1 week of incubation. No significant changes in particle size were observed for the NPs in control conditions or for P1, P2, P3 and P5 in DMEM prior to the 24 h time points. **P1**, mPEG_2000_-(LA)_50_-(TBPC)_50_; **P2**, mPEG_5000_-(LA)_50_-(TBPC)_50_; **P3**, mPEG_4000_-(LA)_50_-(TBPC)_50_; **P4**, mPEG_1014_-(LA)_50_-(TBPC)_50_; **P5**, mPEG_797_-(LA)_50_-(TBPC)_50_.

As can be observed in Figure 1, no substantial changes in size were observed for the NPs kept at 4 °C, confirming primary colloidal stability in these conditions, except for the P4 formulation whereby a slight shift to a larger size was observed over time, indicating aggregation of the NPs. When incubated at 37 °C in DMEM cell culture medium, changes in the DLS plots were apparent after the 24 h time points for all the formulations, with a broadening of the peaks and a shift to larger size ranges, suggesting the particles were aggregating before collapsing. For particles P1, P2, P3 and P5, the DLS traces continued to broaden until the one-week time point. NPs produced from P4 proved to be much less stable than the other formulations, and aggregation of the NPs occurred within a 1 h timeframe. Since standard internalisation assays in cell culture are typically conducted over time periods of several hours, these initial assays indicated that all the NPs except P4 were sufficiently stable for initial cell culture experiments over standard time ranges, and that the formulations were also storage stable.

### 3.2. Cytocompatability

**Figure 2:**
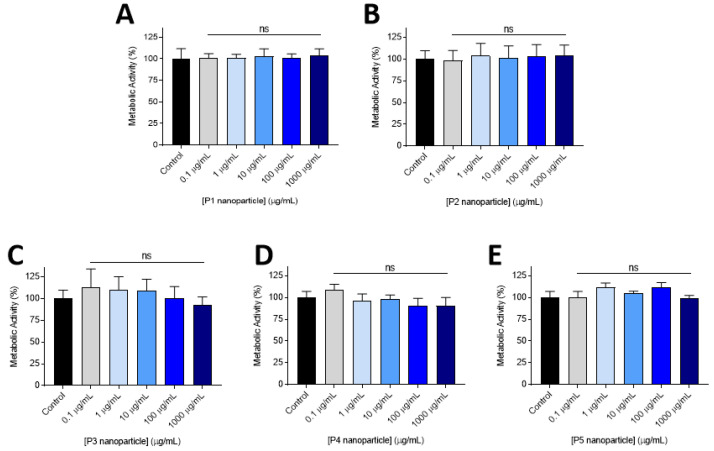
In vitro U87 cell viability assay at 72 h post-treatment with different concentrations of the five NP formulations P1 (**A**), P2 (**B**), P3 (**C**), P4 (**D**) and P5 (**E**) using the PrestoBlue™ metabolic assay. Three independent repeats were carried out (*n* = 4), across three different passages. Error bars show the mean ± SD. [ns: *p* > 0.05, unpaired *t*-test with Welch’s correction, comparing each data set to the control (negative control; medium) data set].

Initial NP cytocompatibility screening was conducted using the PrestoBlue™ assay in the standard U87 glioblastoma-derived cell line. As can be seen from Figure 2, increasing concentrations of each of the polymeric NP formulations tested did not markedly alter metabolic activity compared to the negative control. No observed reduction in cellular metabolic activity was observed after 72 h, indicating that the polymer ‘platform’ of PEG-poly(lactide)-co-poly(carbonate) was well tolerated by the brain tumour cells.

From the initial set of materials, we selected P2 for further in vitro studies, as these polymers formed NPs at a low critical aggregation concentration (CAC = 8.5 µg/mL, exhibited zeta potentials of −23.5 ± 4.5 mV (Supplementary Table S1), suggesting good colloidal stability, and the particle sizes of the as-formed NPs were the equal lowest amongst the set (47 nm), which we anticipated would enhance tissue penetration. The P4 and P5 polymers were omitted due to a lack of stability in storage conditions, while P1 and P3 were formed with PEG chains of reduced length (M_n_ = 2000, 4000) compared to P2 (M_n_ = 5000). It has been reported that increased PEGylation at a surface also aids penetration through brain tissue [33,34], providing further support for P2 as the lead formulation in this context. Accordingly, the P2 polymer was used in further cytocompatibility experiments, and was further derivatised for subsequent experiments by partial side-chain deprotection and coupling of doxorubicin via a urea linker, to form polymer P6-(mPEG_5000_-(LA)_50_-(TBPC)_50−x_-DOX_x_).

### 3.3. Lead Nanoparticle Cytocombability Screening

**Figure 3:**
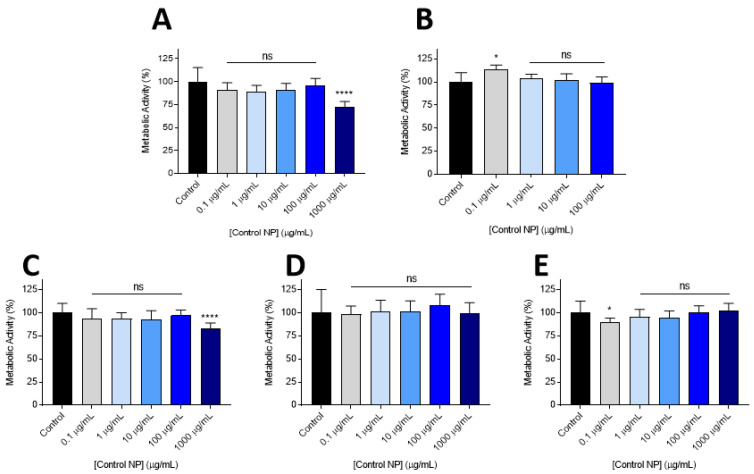
In vitro cell viability following dosing with (0.1–1000 µg/mL) P2 NP formulation for 72 h in human astrocytes (**A**), 9L rat glioma (**B**), GIN-8 (**C**), GIN-28 (**D**) and GIN-31 (**E**) cell lines. Metabolic activity was assessed using the PrestoBlue™ assay. Four independent repeats (*n* = 4) were carried out. Error bars show the mean ± SD. [ns: *p >* 0.05; *: *p* < 0.05; ****: *p* < 0.0001, unpaired *t*-test with Welch’s correction, comparing each data set to the control (negative control; medium) data set].

To demonstrate cytocompatibility of the lead NP candidate in more translationally relevant cell lines, the effects of the polymers on metabolic activity were assessed in a non-tumourigenic, primary human astrocyte line (Figure 3A), 9L rat glioma cells (used widely to generate immune competent glioma allografts) (Figure 3B), and a panel of primary cell lines derived from the invasive margin of GBM patients (Figure 3C–E). These latter cell lines (GIN-8, GIN-28 and GIN-31) better represent the cancerous cells remaining in the brain following maximal safe surgical resection, compared to classical cell lines derived from the tumour core. In these cell lines, P2 demonstrated safe application at all concentrations tested (≤1000 µg/mL) (Figure 3C,D), with the exception of the GIN-8 cell line in which cytotoxicity was observed at 1000 µg/mL (Figure 3E).

### 3.4. Assays to Evaluate Cellular Internalisation of NPs

**Figure 4:**
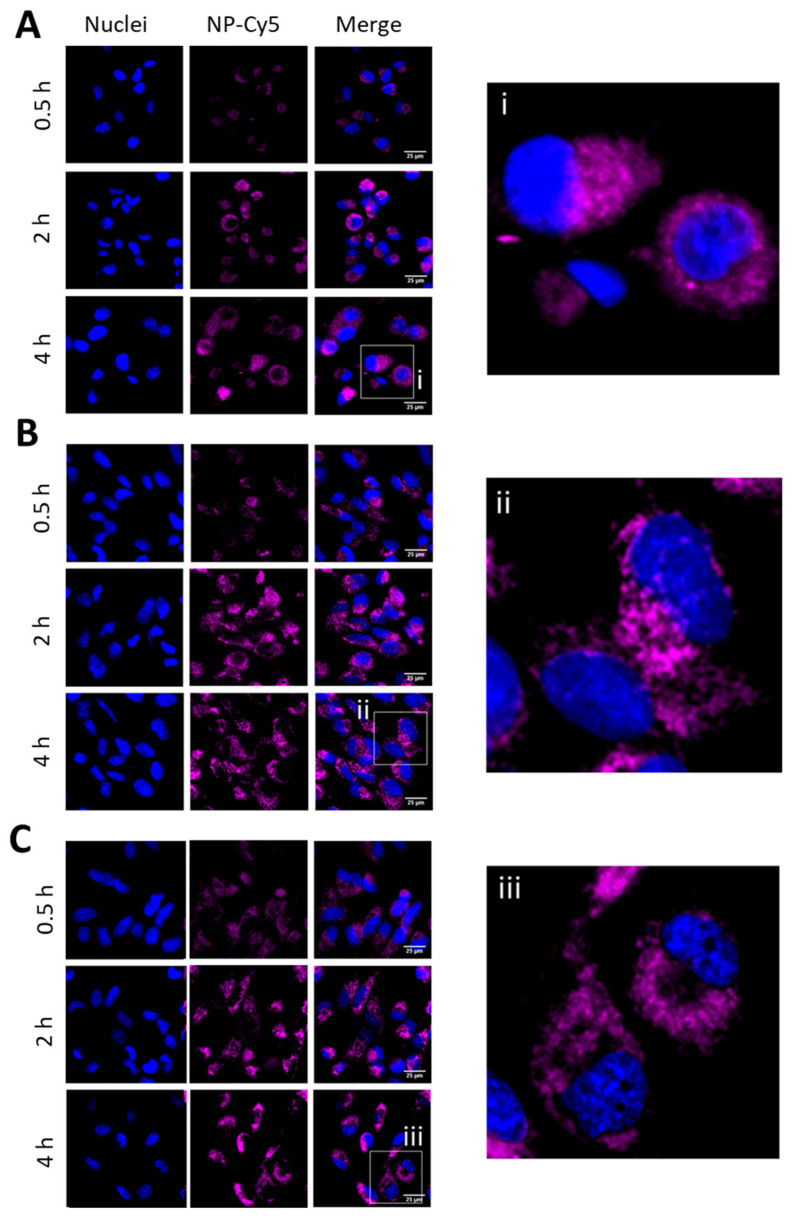
Uptake characteristics of Cy5-NP P2 in GBM cells. (**A**–**C**) Fluorescent micrographs of nanoparticle internalisation in (**A**) U87, (**B**) GIN28 and (**C**) GIN8 cells. Cells were incubated with nanoparticle solution at concentration of 50 µg/mL for 0.5, 2 and 4 h. (i, ii, iii) Enlarged areas highlighted by white boxes. Scale bar = 20 µm. Images are representative of three independent experiments.

In order to deliver any drug cargo, efficient internalisation of NPs is critical, and thus to investigate the uptake of the P2 formulation, a fluorophore (Cy5) was conjugated to the P2 polymer prior to NP formation (see Materials and Methods). These Cy5-NPs were then applied to GIN8, GIN28 and U87 cells for 0.5, 2 and 4 h and the cells imaged via live-cell fluorescence microscopy (Figure 4). The cellular imaging demonstrates that NP uptake appeared time dependent, with the least Cy5 signal being observed at 0.5 h relative to the later time points. Furthermore, from microscopy it can observed that the Cy5-NP signal appears to be localised to the cytoplasm with an absence of signal in the cell nuclei at all time points (Figure 4).

**Figure 5:**
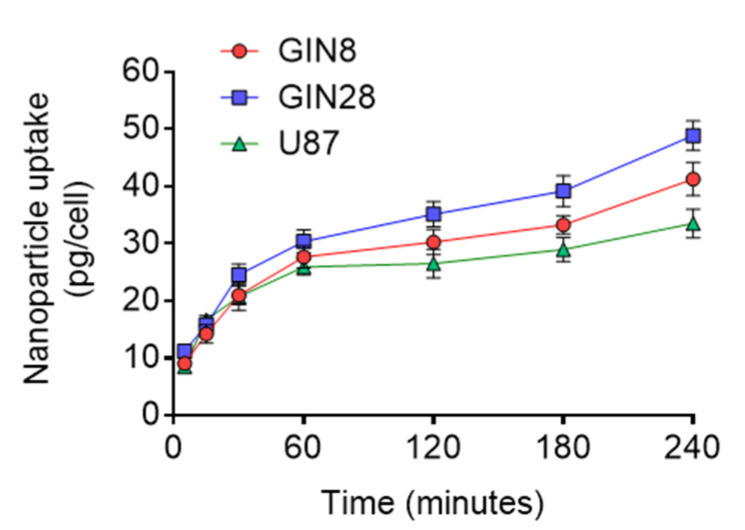
Assessment of NP-Cy5 uptake kinetics in GIN8, GIN28 and U87 cell lines using the fluorometric method. Cy5-NP P2 was applied at 50 µg/mL in 10% (*v*/*v*) containing DMEM. Data presented as the mean ± S.D (*n* = 3).

To investigate the potential differences in the rate of NP uptake in different GBM cell lines, a quantitative kinetic uptake experiment was performed (Figure 5). Analysis reveals that the highest levels of NP uptake were experienced by GIN8 cells, followed by GIN28 cells and with U87 demonstrating the lowest uptake levels. It can be noted that the fastest rates of uptake for all cell lines tested were demonstrated in the first 5–60 min, followed by subsequent rate decrease in the time points thereafter (Figure 5).

**Figure 6:**
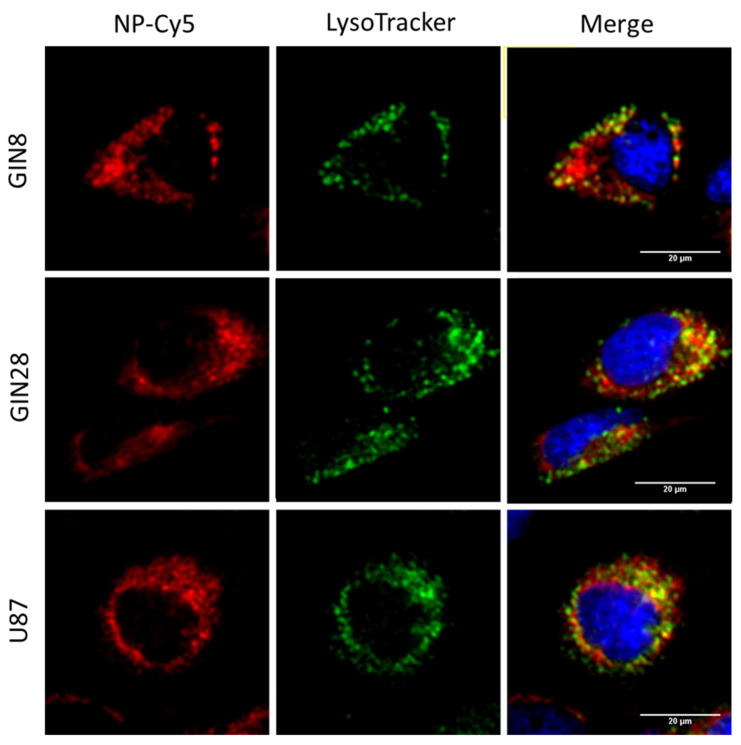
Co-localisation studies in GBM cells. Cells were treated with 50 µg/mL NP-Cy5 for 4 h. Images were processed on Image J software. Cy5 signal of NP-Cy5 was false coloured to red to aid in visualisation of co-localisation with green lysosomal signal. Merged images include NP-Cy5 signal, lysosome stain and Hoechst 33342 staining of nuclei. Scale bar = 20 µm.

Finally, to investigate whether NP-Cy5 P2 localised in lysosomal compartments following uptake, Lysotracker Green DND-26, a lysosomal marker was used in co-localisation studies (Figure 6). Following a 4 h incubation with NPs, co-localisation of NP-Cy5 (red) and Lysotracker green (green) was observed in GIN8, GIN28 and U87 cells, indicating the internalisation of NP-Cy5 P2 was occurring via endocytosis and trafficked to lysosomes. Such intracellular trafficking following endocytosis has been widely reported for polymeric NPs [35,36] and is in agreement with previous studies on our NPs in different cell lines [16,37].

### 3.5. Drug Release Studies

Measurements of DOX concentration following incubation in PBS of the P6 polymer DOX-NP formulation (**P6**-mPEG_5000_-(LA)_50_-(TBPC)_50−x_-DOX_x_), and from the P6 formulation when in the PLGA-PEG microparticulate matrix, were carried out to establish the timeframe of drug release. The PLGA-PEG matrix was prepared in two forms to mimic possible clinical settings. In the first format, a paste was prepared using pre-formed microparticles blended with P6, to represent the formulation being pasted onto the cavity walls of the brain after surgery. In the second form, sintered mixtures of PLGA-PEG and P6 were formed into moulds to mimic brain cavity shaped implants.

**Figure 7:**
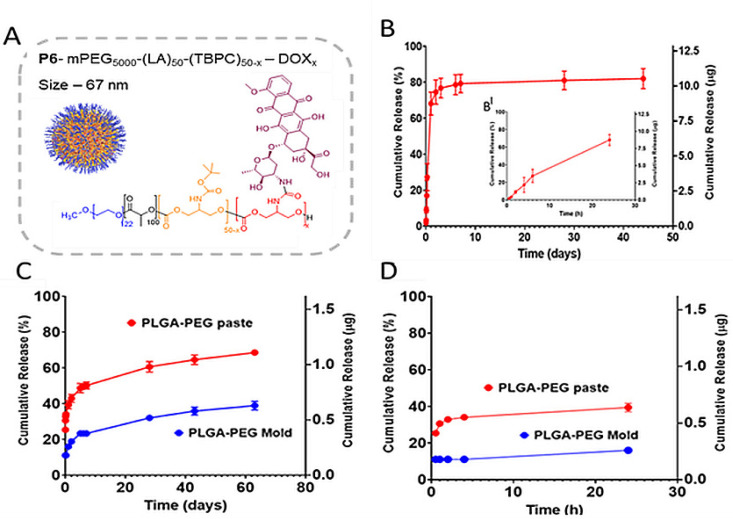
(**A**) P6-mPEG_5000_-(LA)_50_-(TBPC)_50−x_-DOX_x_ formulation, showing average size obtained for each of the in vitro experiments carried out in this body of work. (**B(B^I^))**. Cumulative% and µg of DOX release from the DOX-loaded NPs over 44 days (**B**) and 24 h (**B^I^**). (**C**,**D**). Release was carried out in PBS. Time points represent one independent repeat (*n* = 3) with error bars showing the mean ± one SD. Cumulative% and µg of DOX-NP release from the PLGA/PEG microparticulate matrix over 63 days (**C**) and 24 h (**D**). Release was carried out in PBS. Time points represent one independent repeat (*n* = 3) with error bars showing mean ± one SD.

As apparent in Figure 7, a burst release of DOX from the NPs occurred over the first 6 h (Figure 7B(B^I^)), with over 60% of DOX released within the first 24 h. After this time point, steady state release was observed, with just over 80% of the drug cumulatively released over the following 44 days (Figure 7B). This burst release was surprising, considering the expected stability of the urea bond conjugating the DOX to the polymer backbone [38]. However, it has been reported that a urea bond can be weakened when a large, sterically hindered substituent group constitutes the secondary amine [39,40], and in this case the polymeric backbone may have acted as an appropriately large, sterically hindered moiety. Furthermore, the structures of the kinetically trapped NPs may have influenced the release rate of DOX, as the ingress of aqueous fluids to hydrolyse the polymer-DOX link, is highly dependent on chain packing in the NP. Polymer chains near the surface of the NP would be accessible to the aqueous buffer, leading to more rapid cleavage of urea links, whilst DOX-conjugates trapped near the centre of the particle would be less hydrated, slower to react and thus release the DOX more slowly. In addition, local pH values might vary considerably at different ‘depths’ in the kinetically trapped NP interior, leading to different rates of urea hydrolysis as poly(lactide) bonds also hydrolysed in the NP. Nevertheless, the release profiles obtained indicated that sufficient DOX was released from the NPs within the first 24 h (8.75 ± 1 µg) to achieve concentrations appropriate for in vitro cytotoxicity assessment. In terms of the different formulations, the release from the “paste-like” PLGA/PEG matrix was faster than that from the tightly packed mould. Again, a burst release of the drug-conjugated NPs from the microparticulate matrix paste was observed during the first 8 h. This likely arose because the DOX-NPs remained on the surface of the matrix during the mixing process with the PLGA/PEG microparticles. A much less rapid release profile was obtained from the moulds during the first 24 h, indicating that there were fewer NPs hydrolysing at the surface compared to those in the paste, and the overall level of drug liberated from the moulds was lower than that from the paste. This was probably a consequence of reduced ingress of moisture in the more densely packed matrix. The cumulative release of DOX from the moulds was also less, reaching less than 40% of total drug loaded after two months, whereas almost 70% of DOX was released from the DOX-NPs in the paste over the same time period. For potential formulations used in post-surgery GBM application, DOX release from NPs would be required to occur later than DOX-NP transport from the PLGA/PEG matrix, in order that drug release occurs after NPs have penetrated the brain parenchyma beyond the infiltrative tumour margin, thus potentially targeting distal residual disease cells which persist after surgical resection. These data overall indicated that it was possible to sustain drug release over clinically relevant time points, as well as to effect control over burst vs. sustained release.

### 3.6. Effects of DOX-Loaded Nanoparticles in Glioblastoma Cells

**Figure 8:**
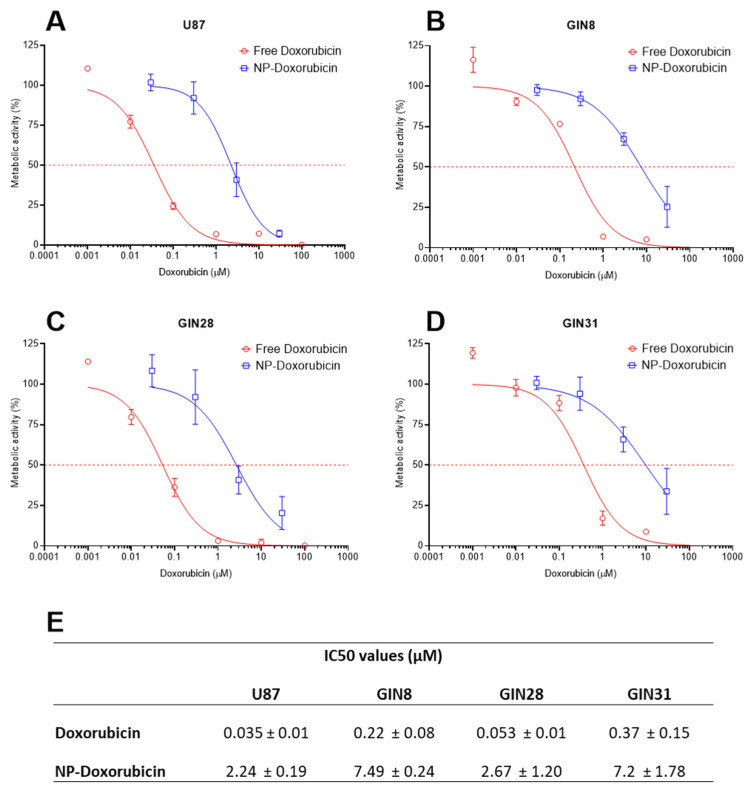
Potency of DOX and DOX-NPs in U87 (**A**), GIN-8 (**B**), GIN-28 (**C**), and GIN-31 (**D**) cell lines. Treatments were applied in DMEM containing 10% FBS and exposed for 72 h. Potency was determined via assessment of cellular metabolic activity using the PrestoBlue assay. Data presented as the mean ± S.D from four independent repeats. IC_50_ values (mean ± S.E.M) were calculated and are shown in the table (**E**).

The potencies of free DOX and DOX-NPs were assessed in U87 cells and in three primary GBM cell lines (GIN-8, GIN-28 and GIN-31) (Figure 8). In both treatment conditions (DOX and DOX-NP), it was observed that the commercially available U87 cell line was the most treatment-sensitive GBM line tested. The GIN-28 cells were determined to be the most susceptible primary patient-derived cell lines to DOX-based treatments, followed by GIN-8 and GIN-31, which displayed similar levels of sensitivity to DOX and DOX-NP (Figure 8). The variation in potency observed between the patient-derived lines is likely due to the variability between patients (intertumour heterogeneity) and highlights the importance of studying multiple cell lines. Furthermore, the relatively lower sensitivity to DOX in GIN lines likely reflects the lower proliferative rate of primary patient-derived cells lines, relative to the in vitro-adapted U87 line. IC_50_ in GIN lines are therefore a more physiologically accurate test bed to evaluate chemotherapy response.

The experiments demonstrated that the DOX-NPs were less cytotoxic than free DOX in all cell lines tested, with IC_50_ values 70-fold, 34-fold, 50-fold and 20-fold higher in U87, GIN-8, GIN-28 and GIN-31 cells, respectively (Figure 8E). Interestingly, the decreases in potency experienced by the pro-drug formulation in the different cell lines (Figure 8E) reflect the rankings of the internalisation levels in the tested cell lines (Figure 5). For example, U87 cells demonstrated the lowest levels of internalisation and subsequently the largest decrease in DOX vs DOX-NP potency.

While the decrease in drug potency is not unexpected over the time periods of 2D cell culture assays, as internalisation of free drug is more rapid than endocytosis and pro-drug activation from a polymer chain, in previous studies these polymer DOX-NPs achieved greater than or equal potency to free DOX in breast, lung, skin and intestinal tumour cells [16,37]. We suggest therefore that the specific GBM cells studied, either internalised the NPs less efficiently than the free drug over the time periods of the assay and/or that the urea linker in the DOX polymer conjugates was more stable in these cell lines than in those assayed previously.

Nevertheless, the demonstration of appropriate cytotoxicity in patient-derived GBM cells with the DOX-NPs, together with their successful incorporation in the PLGA/PEG microparticulate local delivery matrix (Figure 8) and successful release of DOX, highlights the potential of this hybrid system for future development in GBM pre-clinical models.

## 4. Conclusions

We have investigated the effects of a range of polymer NPs in GBM cell lines from the therapy relevant infiltrative margin and found that all polymeric formulations were well tolerated in the absence of a drug payload. A single polymer formulation (P2) was selected for further study based on an expected favourable profile of particle size, zeta potential and colloidal stability. These studies included investigating the metabolic activity of a primary human astrocyte cell line and a panel of primary GBM cell lines when treated with both P2 NPs and a conjugate of P2 with DOX, termed P6. Control P2 NPs exhibited no adverse effects at concentrations of up to 100 µg/mL in the astrocyte line and in each of the primary GBM cell lines, whereas P6 DOX-NPs were cytotoxic for U87 cells and the three primary GBM cell lines investigated. Drug release studies were carried out using P6 DOX-NPs in buffer and P6 DOX-NPs loaded into PLGA/PEG paste and moulded monoliths. Data from these experiments indicated that sufficient DOX was released from the NPs to achieve a cytotoxic dose in vitro. Furthermore, a burst release of the drug from the P6 DOX-NPs in the microparticulate paste matrix (mimicking the intended surgical application) was observed during the first 8 h, whereas a slower release occurred from the moulds. In both the paste and mould formulations a sustained release profile was observed after 24 h and up to 2 months, and for the paste ~70% of DOX was released after one month. These release profiles are appropriate for drug therapies required in GBM patients after surgical resection of primary tumours and warrant translational assessment using human orthotopic xenograft models for post-surgical drug delivery.

## Figures and Tables

**Table 1:** Chemical characterisation of the PEGylated copolymers, nature of PEG blocks and number of polymer arms.

Polymer	PEG Length (Da)	Final Polymer Arms	M_n_ (NMR)	M_n_^1^ (GPC)	Ð
**P1.** mPEG_2000_-(LA)_50_-(TBPC)_50_	2000	1	20,500	20,400	1.3
**P2.** mPEG_5000_-(LA)_50_-(TBPC)_50_	5000	1	23,000	24,000	1.2
**P3.** PEG_4000_-(LA)_50_-(TBPC)_50_	4000	2	21,000	20,000	1.1
**P4.** PEG_1014_-(LA)_50_-(TBPC)_50_	1014	3	19,000	16,400	1.2
**P5.** PEG_797_-(LA)_50_-(TBPC)_50_	797	4	21,000	16,300	1.2

^1^ Compared to PMMA standards, Ð: dispersity.

## Data Availability

Data is available upon request from the corresponding author.

## Supplementary Materials

The following are available online at https://www.mdpi.com/1999-4923/13/2/208/s1, Figure S1. Effect of culture time and seeding density on PrestoBlue fluorescence; Table S1. NP size and zeta potentials.
