# Supplementary material for: Polymer Pro-Drug Nanoparticles for Sustained Release of Cytotoxic Drugs Evaluated in Patient-Derived Glioblastoma Cell Lines and In Situ Gelling Formulations
Source: Pharmaceutics. 2021 Feb 3;13(2):208. doi: 10.3390/pharmaceutics13020208 (PMC7913572; doi:10.3390/pharmaceutics13020208)
Supplement: Supplementary file 1 [file pharmaceutics-13-00208-s001.pdf]

pharmaceutics

MDPI

Supplementary Materials: Polymer Pro-Drug Nanoparticles for Sustained Release of Cytotoxic Drugs Evaluated in Patient-Derived Glioblastoma Cell Lines and In Situ Gelling Formulations

Catherine E. Vasey, Robert J. Cavanagh, Vincenzo Taresco, Cara Moloney, Stuart Smith, Ruman Rahman and Cameron Alexander

Citation: Vasey, C.E.; Cavanagh, R.J.; Taresco, V.; Moloney, C.; Smith, S.; Rahman, R.; Alexander, C. Polymer Pro-Drug Nanoparticles for Sustained Release of Cytotoxic Drugs Evaluated in Patient-Derived Glioblastoma Cell Lines and In Situ Gelling Formulations. *Pharmaceutics* **2021**, *13*, 208 https://doi.org/10.3390/pharmaceutics13020208

Academic Editor: Francesca Greco, Az Alddien Natfji
Received: 4 November 2020
Accepted: 1 February 2021
Published: 3 February 2021

Publisher's Note: MDPI stays neutral with regard to jurisdictional claims in published maps and institutional affiliations.

Copyright: © 2021 by the authors. Submitted for possible open access publication under the terms and conditions of the Creative Commons Attribution (CC BY) license (http://creativecommons.org/licenses/by/4.0/).

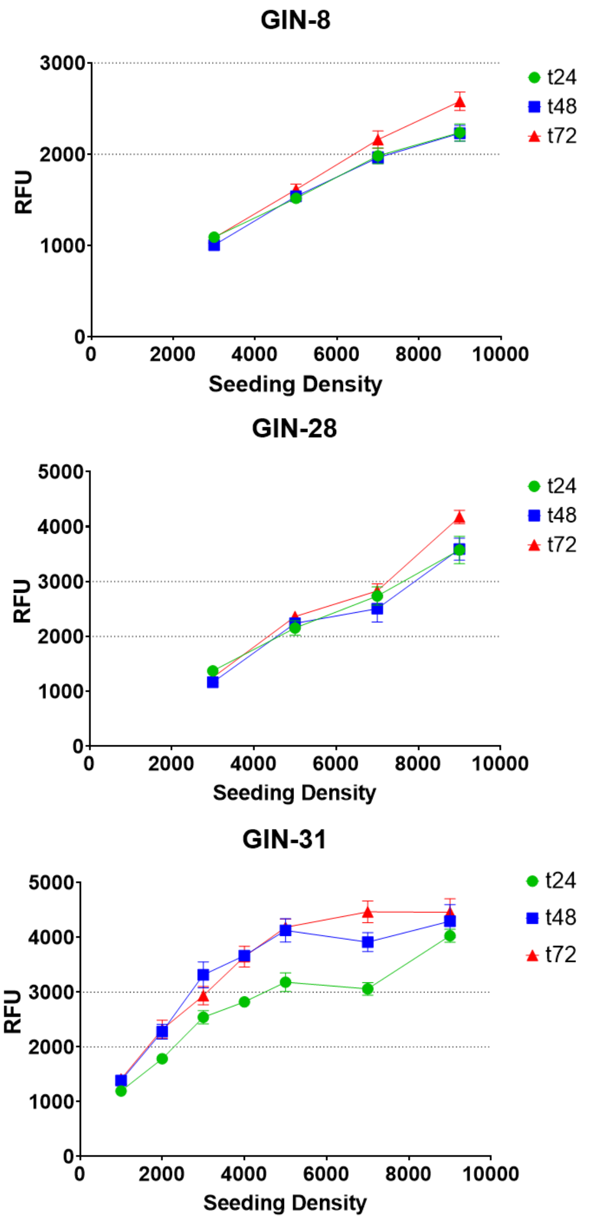

Figure S1. Effect of Culture Time and Seeding Density on PrestoBlue Fluorescence. Cell seeding density experiment for the PrestoBlue™ assay at 24, 48 and 72h. Four independent repeats were carried out (n = 4) for the GIN-8 line and two independent repeats (n = 4) were carried out for the GIN-28 and GIN-31 cell lines. Error bars show mean ± S.E.M.

Table S1. NP size and zeta potentials. Average values from at least three sample replicates produced from three batches: measurements in MilliQ water.

Polymer	Size (nm)*	PDI	Z-potential(mV)	CAC
**P1.** mPEG$_{2000}$-(LA)$_{50}$-(TBPC)$_{50}$	68.6 ± 1.1	0.1 ± 0.0	−26.9 ± 2.5	9.5
**P2.** mPEG$_{5000}$-(LA)$_{50}$-(TBPC)$_{50}$	61.4 ± 1.0	0.1 ± 0.0	−23.5 ± 4.5	8.5
**P3.** PEG$_{4000}$-(LA)$_{50}$-(TBPC)$_{50}$	68.1 ± 1.9	0.1 ± 0.0	−10.7 ± 3.5	12.7
**P4.** PEG$_{1014}$-(LA)$_{50}$-(TBPC)$_{50}$	118.9 ± 3.8	0.1 ± 0.0	−29.2 ± 5.5	11.5
**P5.** PEG$_{797}$-(LA)$_{50}$-(TBPC)$_{50}$	119.6 ± 3.1	0.1 ± 0.0	−30.1 ± 5.5	12.1

* at 1mg/mL.
